# Small bowel obstruction in adults, Ladd's band is an exceptional cause: a case report

**DOI:** 10.11604/pamj.2024.47.34.36435

**Published:** 2024-01-26

**Authors:** Jaouad Naddouri, Rachid Khouah, Hamza Sekkat, Younes Bakali, Mouna M'hamdi EL Alaoui, Mohamed Raiss, Farid Sabbah, Abdelmalek Hrora

**Affiliations:** 1 Department of Digestive and General Surgery, Department of Surgery C, University Hospital Center Ibn Sina, Rabat, Morocco,; 2 Department of Digestive and General Surgery, Department of Surgery A, Lucien Hussel Hospital Center, Vienne, France

**Keywords:** Intestinal volvulus, small bowel, Ladd’s band, laparotomy, case report

## Abstract

Malrotation of the gut is a congenital anomaly of foetal intestinal rotation and it's principally discovered in early childhood as acute intestinal obstruction. This condition is veritably rare and constantly silent in adults. Intestinal malrotation in adults is frequently asymptomatic and is diagnosed as a casual finding during a radiological examination performed for other reasons. Infrequently, it can be diagnosed in adults, associated with an acute abdomen. Adult patients rarely present with acute midgut volvulus or internal hernias caused by Ladd's bands. We present a case of an admitted 18-year-old female with a small bowel obstruction due to an intestinal volvulus complicating intestinal malrotation in the presence of Ladd's band. Laparotomic Ladd's procedure was performed successfully with division of Ladd's band, adhesiolysis, appendicectomy, and reorientation of the small bowel on the right and the cecum and colon on the left of the abdominal cavity; the postoperative evolution was favorable. Although it is a rare pathology, it should be kept in mind in cases of patients presenting small bowel obstruction.

## Introduction

Intestinal malrotation can be described as a congenital anomaly of the intestinal rotation and fixation at some stage in the improvement of the foetus. This anomaly is caused by partial or complete failure of 270-degree counterclockwise rotation of midgut around superior mesenteric vessels in foetal life [1]. These rotation anomalies can lead to complications, sometimes life-threatening events, which usually occur during the period of neonatal or pediatric age. The fact that this pathology is exceptional in adults and that its symptomatology is quite variety can be the source of many errors and delays in diagnosis and therapeutic [2]. Ladd first described the procedure to treat malrotation and volvulus in 1932 and since then it has been the definitive treatment for intestinal malrotation [3,4]. We present a case of an 18-year-old female admitted for small bowel obstruction due to an intestinal volvulus complicating an intestinal malrotation in the presence of Ladd´s band, operated in emergency for which we performed a Ladd procedure. The postoperative evolution was favorable.

## Patient and observation

**Patient information:** an 18-year-old woman, without any particular pathological history, presented to the emergency department of the Ibn Sina Hospital (Rabat, Morocco), with a three-day history of abdominal distension. She also complained of flatus and a three-day incapacity to defecate.

**Clinical findings:** the clinical exam revealed vital signs within normal range. The abdominal region looked distended. Auscultation bowel sounds had been reduced, and hyper resonant sounds were positive on percussion. A digital rectal exam revealed an empty trunk.

**Timeline of current episode:** there was a history of intermittent constipation and abdominal pain in the past two years.

**Figure 1:**
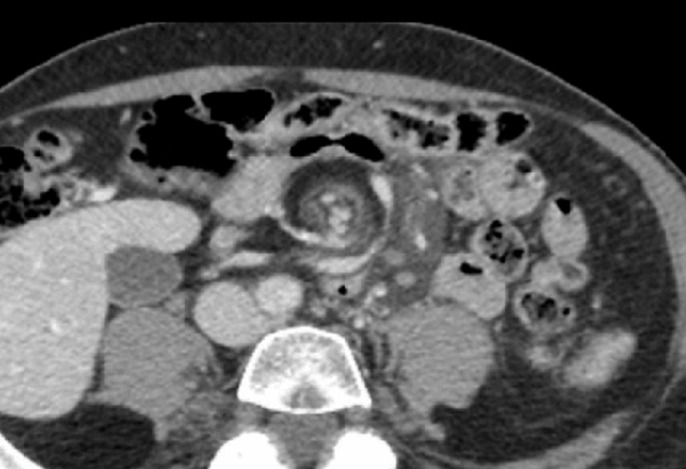
axial abdominal scan showing the whirl sign

**Diagnostic assessment:** blood count was normal, biochemical studies showed slightly increased urea and creatine. The abdominal scan showed dilatation of the small bowel in addition to the whirl sign, without any signs of intestinal necrosis. The radiologist suggested a primary diagnosis of intestinal volvulus in combination with incorrect intestinal rotation (figure 1).

**Figure 2:**
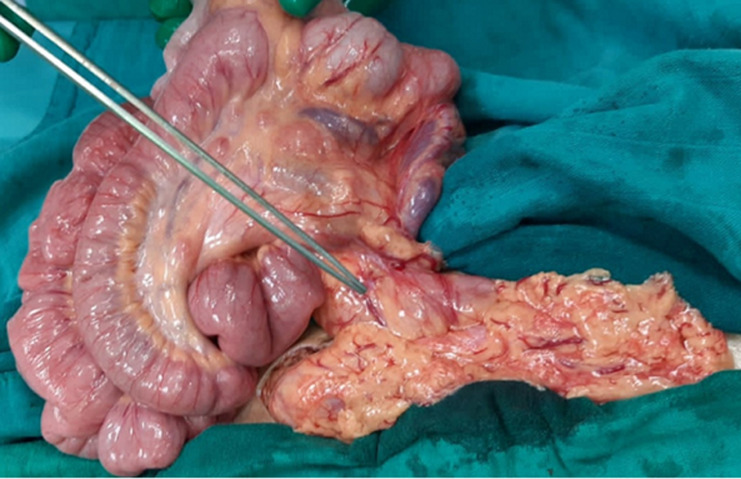
intraoperative image showing the intestinal and mesenteric volvulus

**Figure 3:**
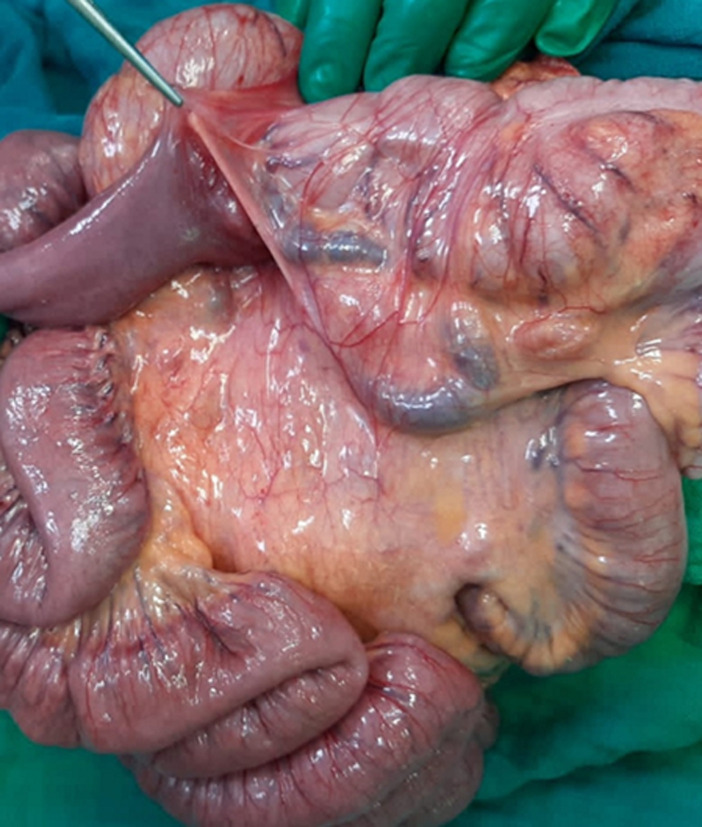
intraoperative image representing the fibrous band after detorsion and small bowel adhesiolysis

**Figure 4:**
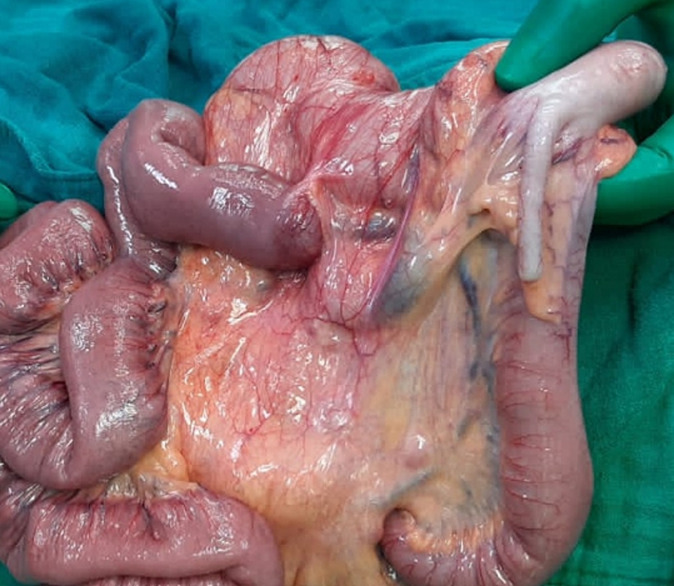
intraoperative image showing intestinal malrotation

**Figure 5:**
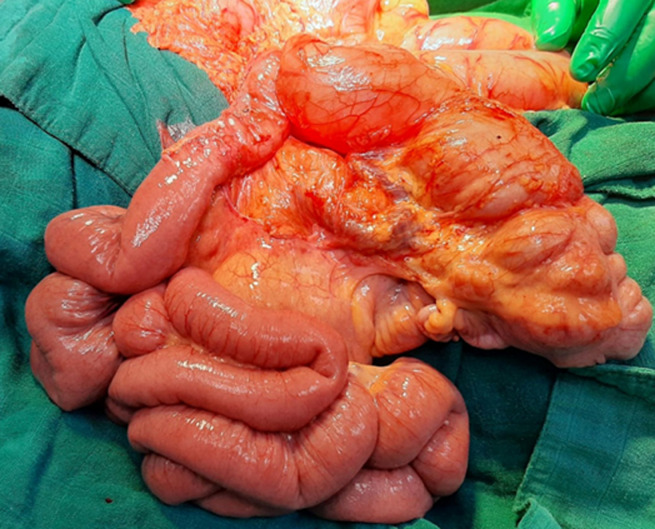
reorientation of the small bowel on the right and the cecum and colon on the left after appendicectomy

**Therapeutic interventions:** emergency surgery was performed, with an exploratory laparotomy. This laparotomy was set up an intestinal and mesenteric volvulus (figure 2), many visceral adhesions and Ladd´s band (figure 3), in addition to an intestinal malrotation (figure 4). Ladd's procedure was carried out to alleviate bad intestinal rotation. The method entails counterclockwise diversion of the bowel, surgical division of Ladd's bands, widening of the small intestine mesentery, acting an appendectomy, and reorientation of the small bowel on the proper and the colon at the left of the abdominal cavity (figure 5).

**Figure 6:**
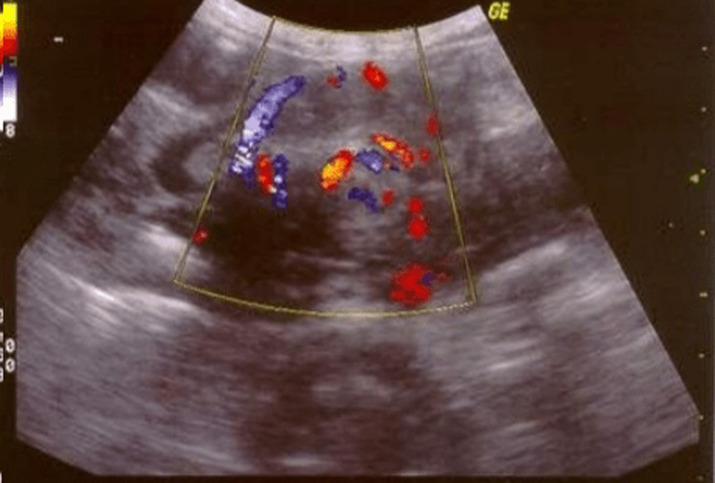
ultrasound Doppler showing malrotation vessels

**Follow-up and outcome of interventions:** the patient recovered successfully and was released on day two post-op. There was no recurrence of symptoms after a one-year follow-up.

**Patient perspective:** the patient provided her perspective on the treatment, stating that she is not experiencing any symptoms and may return to normal activity.

**Informed consent:** the patient consented with knowledge to the release of his clinical data. The presented facts are anonymized, and the threat of identification is minimum.

## Discussion

The normal development of the mid-gut occurs in three stages of the rotation process between the 4^th^ and 12^th^ week of the embryonic period. During this phase, the duodenum is attached retro-peritoneally to the left Treitz ligament and the caecum at the bottom of the right abdomen. In case of malrotation, a peritoneal fibrous tape, also known as the Ladd tape, can compress the duodenum and cause duodenal obstruction. Intestinal malrotation is a disease of the newborn, as it frequently manifests in the first month of life; an adult manifestation is very rare. The clinical profile of adults is more variable and may be asymptomatic. Adult patients seldom have acute intestinal volvulus or internal hernias caused by Ladd bands [5-7]. Prevalence in adults is estimated to be 0.2% [8]. Diagnosis in adults is challenging because of the low incidence. Ultrasound Doppler allows to suspect an intestinal malrotation when the mesenteric vein is located to the left of the superior mesenteric artery on cross-sections of the epigastrium (figure 6) [9]. It is important to assess these vascular ratios as low as possible under the spleno -mesaraic confluence, which is not always possible when air distension is significant in this area [10,11].

However, according to recent literature data, is an abdominal scan with injection and high opacity which is today the examination of choice for the diagnosis of intestinal volvulus due to intestinal malrotation in adults [12,13]. The main specific sign is referred to as "Whirl". Other signs may be shown as duodenal blockage, ischemia of the superior mesenteric vessels and intestinal malrotation itself (Treitz angle to the right of the spine, lack of passage of the duodenum in the aorto-mesenteric clamp and reversal of the mesenteric vessels at their origin). The Ladd procedure remains the basic treatment for volvulus on rotational abnormality in adults and children. This procedure consists of a reduction of the volvulus, followed by a setting in complete common mesentery of the small intestine to avoid any recurrence of the volvulus. Then comes the time of an appendectomy. The appendix should be systematically removed to prevent episodes of acute appendicitis in the ectopic position [14,15].

## Conclusion

Intestinal malrotation is an entity of low incidence, and adult presentation is even rarer. Some cases are asymptomatic. In symptomatic cases, there are two awesome patterns of person presentations: acute and chronic. Acute presentation is extra uncommon and may be because of volvulus of the midgut or ileocaecum, said because the maximum not unusual motive of bowel obstruction in adults with intestine malrotation.

## References

[1] Agha RA, Borrelli MR, Farwana R, Koshy K, Fowler AJ, Orgill DP (2018). The SCARE Statement: updating consensus surgical Case REport (SCARE) guidelines. Int J Surg.

[2] Peycelon M, Kotobi H (2012). Complications des anomalies embryologiques de la rotation intestinale: prise en charge chez l'adulte. EMC Techniques chirurgicales-Appareil digestif.

[3] LADD WE (1932). Congenital obstruction of the duodenum in children. N Engl J Med.

[4] LADD WE (1936). Surgical diseases of the alimentary tract in infants. N Engl J Med.

[5] von Flüe M, Herzog U, Ackermann C, Tondelli P, Harder F (1994). Acute and chronic presentation of intestinal non rotation in adult. Dis Colon Rectum.

[6] Durkin ET, Lund DP, Shaaban AF, Schurr MJ, Weber SM (2008). Age related differences in diagnosis and morbidity of intestinal malrotation. J Am Coll Surg.

[7] Pickhardt PJ, Bhalla S (2002). Intestinal malrotation in adolescents and adults: spectrum of clinical and imaging features. AJR Am J Roentgenol.

[8] Gamblin T, Stephens R, Johnson R, Rothwell M (2003). Adult malrotation: a casreport and review of the literature. Curr Surg.

[9] Rafaa S (2010). Volvulus du grêle sur malrotation intestinal a propos de 36 cas.

[10] Orzech N, Navarro OM, Langer JC (2006). Is ultrasonography a good screening test for intestinal malrotation?. J Pediatr Surg.

[11] Lambot K, Lougue-Sorgho LC, Gorincour G, Chapuy S, Chaumoitre K, Bourlière-Najean B Les urgences abdominales non traumatiques de l’enfant.

[12] Durkin ET, Lund DP, Shaaban AF, Schurr MJ, Weber SM (2008). Age-related differences in diagnosis and morbidity of intestinal malrotation. J Am Coll Surg.

[13] Pickhardt PJ, Bhalla S (2002). Intestinal malrotation in adolescents and adults: spectrum of clinical and imaging features. AJR Am J Roentgenol.

[14] Orzech N, Navarro OM, Langer JC (2006). Is ultrasonography a good screening test for intestinal malrotation?. J Pediatr Surg.

[15] Israelit S, Brook OR, Nira BR, Guralnik L, Hershko D Left-sided perforated acute appendicitis in an adult with midgut malrotation: the role of computed tomography.

